# Temporal Pattern and Age-Period-Cohort Analysis of Breast Cancer Incidence in Iranian Women (2009–2017)

**DOI:** 10.34172/aim.2023.44

**Published:** 2023-06-01

**Authors:** Marzieh Eslahi, Gholamreza Roshandel, Narges Khanjani

**Affiliations:** ^1^Department of Biostatistics and Epidemiology, School of Public Health, Kerman University of Medical Sciences, Kerman, Iran; ^2^Golestan Research Center of Gastroenterology and Hepatology, Golestan University of Medical Sciences, Gorgan, Iran; ^3^Neurology Research Center, Kerman University of Medical Sciences, Kerman, Iran

**Keywords:** Age-period-cohort, Breast cancer, Incidence, Iran

## Abstract

**Background:**

Breast cancer accounted for 28.1% of all female cancers in 2020 in Iran. This study was conducted to evaluate the time trend of breast cancer incidence and to identify the changes of breast cancer incidence in age, period, and birth cohorts in Iran, in the 2009–2017 timeframe.

**Methods:**

Annual cancer statistics for female breast cancer were obtained from the Iranian National Population-based Cancer Registry (INPCR) database from 2009 to 2017. The age-period-cohort (APC) analysis was used to evaluate the time trend of breast cancer incidence in age, period and birth cohorts between 2009 and 2017. R package (Epi) was used to analyze data. Results were considered statistically significant at *P*<0.05.

**Results:**

The age effect showed an increased incidence of breast cancer until the age of 45, and after this age the speed of increase was slower until 65 years. There was an increased diagnosis in 2015–2017 (period effect) for many age groups, especially in the 70- and over 80-year-old group.

**Conclusion:**

Our findings indicated that breast cancer incidence peaks in the age of 45 in Iranian women, which is a decade earlier compared to the Western world. The period effect in 2015–2017 can be explained by the fact that in 2014, the former Iranian pathology-based cancer registry was upgraded to a population-based cancer registry, which resulted in improved coverage of cancer registry and case finding.

## Introduction

 Approximately 18.1 million new cancer cases (excluding non-melanoma skin cancer) and almost 9.9 million cancer deaths (excluding non-melanoma skin cancer) occurred in 2021 worldwide.^1^ In the same year, breast cancer with 2,261,419 new cases accounted for 11.7% all cancers and with an ASR of 47.8 per 100 000 was the most common cancer worldwide.^1^ The incidence and mortality rates of breast cancer vary remarkably across geographic regions.^2^ Incidence rates are 88% higher in developed countries than in developing countries, with the highest incidence rates found in Australia, New Zealand, Western Europe, Northern America, and Northern Europe and the lowest rates in central America, eastern and middle Africa, and south central Asia.^1^ Developing regions have faced cancer transitions and changing profiles of common cancer types.^3^ Iran, as a developing country, has faced population aging and an increase in cancer risk factors during the recent decade.^4^ Regional reports have shown a rising trend for the incidence rate of different cancers, especially breast cancer.^4^ According to results of the Iranian National Population-based Cancer Registry (INPCR) in 2014, breast cancer accounted for 25% of all female cancers and was the most commonly diagnosed malignancy among women with an ASR of 34.53 per 100 000.^4^

 Long-term data from vital sources enable us to quantify the temporal changes in incidence rates over time and may help detect the major risk factors. Trend analyses may also suggest novel hypotheses or confirm the existing ones.^5^ Traditionally, trend analysis of cancer incidence is often performed through age-adjusted rates and calendar time, while the other effects are ignored.^5,6^ The generation variable is important in studying the changes of cancer incidence in societies, and can be evaluated in age-period-cohort (APC) analysis. In recent decades, temporal analyses broken down by a third component, the birth cohort, have become a conventional and standard mode of analysis.^5^ Cohort effects are characterized as the environmental exposures in prenatal period or early in life, and reflect factors shared by members of the same group with the same age.

 Period effects are defined as a prompt change in the incidence rates of all age groups (regardless of their birth cohorts), and reflect events that rapidly change all age-specific rates with the same order of magnitude.^7^

 The analysis of the incidence of disease by age neglecting the cohort and period effects may lead to incorrect conclusions.^8^ APC models identify changes in rates of cancer incidence and mortality over time that may be attributable to age, time period of diagnosis, and birth cohort.^9^ To the best of our knowledge, no age, period or birth analysis has been done on breast cancer incidence in Iran, so far. This study was conducted to identify the changes of breast cancer incidence in age, period, and birth cohorts in Iranian women, in the 2009–2017 timeframe.

## Materials and Methods

 This is a longitudinal study based on all cases of female breast cancer recorded in the INPCR database from 2009 to 2017. We obtained unidentifiable cancer data from the INPCR database. A population-based cancer registry (PBCR) records all the new cases of cancers in a defined population and in a defined time period.^10^ Indeed, all breast cancer cases occurring during 2009-2017 were included in the analysis.

 The INPCR database includes data on type of cancer, topography, morphology, gender, and age, date of diagnosis, and place of residence. All INPCR data are coded according to the International Classification of Diseases- Oncology 3^rd^ Edition (ICD-O-3). Breast cancer is identified with the topographic codes C50.0-50.9. Since age data was continuous, we categorized age data in 9 age groups (0-9, …, 70-79, and 80 ^+^ ).

 We also obtained population data based on the national census from the website of the Statistical Center of Iran.^11^

 We calculated the annual age-specific rates per 100 000 for 10-year age groups (0-9, …, 70-79, and 80 ^+^ ), by diving the number of breast cancer cases in each age group by the total population in each age group.

 We performed an APC analysis to determine the effects of age, period, and birth cohort on the temporal trend of breast cancer incidence in the study period (2009-2017). The period was subdivided into 3-year intervals (2009-2011, 2012-2014, and 2015-2017). We calculated birth cohorts by differing age from period (Cohort = Period–Age). The APC regression model follows a Poisson distribution and is shown with the following equation:

 Yij = µ + αi + βj + γk + εij,

 i = 1,..., a,

 j = 1,..., p,

 k = i + j- 1.

 where the response variable Yij, is incidence in cell (i, j) in the i-th row and j-th column. µ is the intercept and εij is random errors that are defined for each year and age group. αi, βj, and γk are the i-th age, j-th period and k-th cohort effects, respectively.^12^ However, the collinearity among the age, period, and cohort has been shown to result in identification problems in model parameter estimates^12^; therefore, we used the intrinsic estimator in the R function apc.fit of Epi package in R (version 4.0.4). The goodness-of-fit for models were determined by deviance. Results were considered significant at *P* < 0.05.

## Results

**Figure 1:**
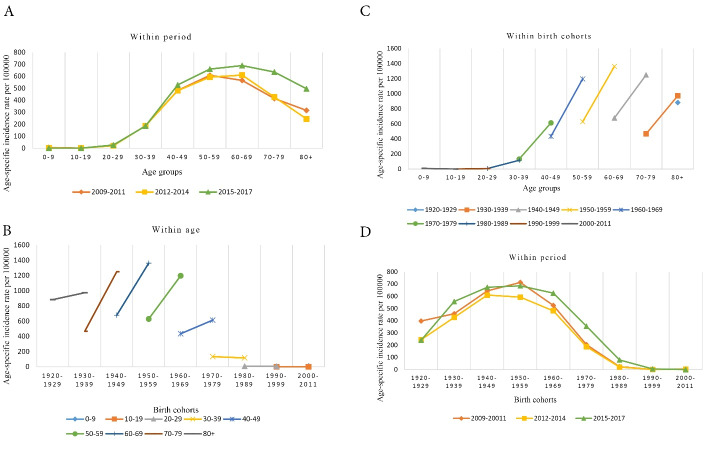
Distribution of Breast Cancer Incidence in Iranian Women from 2009 to 2017. (A) Age-period distribution of breast cancer incidence in Iranian women during 2009–2017. (B) Cohort-age distribution of breast cancer incidence in Iranian women during 2009–2017. (C) Age-cohort distribution of breast cancer incidence in Iranian women during 2009–2017. (D) Cohort-period distribution of breast cancer incidence in Iranian women during 2009–2017

 During 2009–2017, there were 110,588 cases of women breast cancer recorded in the INPCR. The mean age of women at the time of diagnosis was 51.08 ± 12.61. Breast cancer was more prevalent in the age groups 40-49 and 50-59. Figure 1 shows the distribution of breast cancer incidence in Iranian women from 2009 to 2017 using age and period indices.

 The age-period graph (A) shows that in all three periods the incidence rate of breast cancer started to increase in the 30-39 age group. In 2009-2011, breast cancer incidence reached its peak in the 50-59 age group, and then decreased. In 2012-2014 and 2015–2017, breast cancer incidence rate reached its peak in the 60-69 age group, and then decreased.

 The cohort-age graph (B) shows that in age groups above 40, older birth cohorts experienced a lower incidence. The highest incidence rate was observed in women of the age group 60–69 in the 1950–59 birth cohort.

 The age-cohort graph (C) shows that the highest incidence rates of breast cancer were observed in women of the 1960, 1950 and 1940 birth cohorts, who were in their 40, 50s and 60s.

 The cohort-period graph (D) shows that in 2009-2011 and 2015–2017, the highest incidence rate of breast cancer was observed in women in the 1950 birth cohort. But in 2012–2014, the highest incidence rate of breast cancer was observed in women in the 1940 birth cohort.

**Figure 2:**
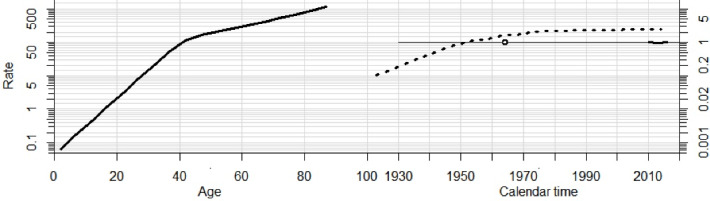
Effects of Age, Period and Birth Cohort on the Trend of Incidence Rate of Breast Cancer in Iranian Women from 2009–2017

Figure 2 shows the APC effect on the incidence of women breast cancer from 2009-2017 in Iran using a multiple regression model. In the age affect (solid line in the far left), after adjustment for period and cohort indices, as age increased, the incidence rate of breast cancer increased. The incidence rate increased sharply with age until the age of 45, then the rising trend became slower after the age of 45.

 In the birth cohort effect (the dotted line in the middle), after adjustment for age and period indices, the incidence rate of breast cancer increased, as far as the women born before 1950, after which it had a stable trend until 1980. Afterwards, it started to decrease in more recent birth cohorts.

 In the period effect (short line in the far right), after adjustment for age and birth cohort indices, we observed that the incidence rate started to decline from 2009 to 2013. Then it started to increase until the end of the study period (2017).

 With regard to the model deviance in Table 1, the full model (APC model) is the best model to explain the data.

**Table 1:** Goodness-of-Fit of Age-Period-Cohort Model Assessment for Breast Cancer Incidence Rates in Iran, 2009–2017.

**Model**	**Model Deviance**	**Model df**	**Test Deviance**	**Test df **	***P***** Value**
Age	2848.16	Ref	Ref	Ref	Ref
AP	2460.42	45	307.18	5	< 0.001
AC	2254.73	41	296.95	5	< 0.001
APC	2153.24	40	101.49	1	< 0.001

df, degree of freedom

## Discussion

 Epidemiological data on cancer has a pivotal role in cancer control planning.^4^ The PBCR is an essential tool for providing data for planning and development of cancer control programs.^13^

 The incidence rate of breast cancer has had a rising trend in Middle Eastern countries, including Iran. In Asia, the incidence of breast cancer peaks among premenopausal women aged 40–49, whereas in developed countries, breast cancer is predominantly a disease of postmenopausal women.^14-16^ In Iran, the mean age range of breast cancer diagnosis in women is between 40–50 years, which is about 10–15 years younger than the global average, and noticeably lower than patients in Western countries.^17,18^

 Our APC analysis showed that the incidence rate of breast cancer in Iranian women increased sharply with increasing age, with a peak in the age of 45. After the age of 45, the speed of increase was slower. We also observed a period effect which can be explained, to some extent, by the fact that upgrades in the national cancer registry from pathology-based to population-based resulted in improved coverage and completeness of the national cancer registry records.

 Our findings indicated that in birth cohorts, the incidence rates increased for women born before 1950, after which the incidence rates were lower in more recent birth cohorts. Almasi-Hashiani et al suggested that postmenopausal status, older age at marriage, lower duration of breastfeeding, and use of oral contraceptive may increase the risk of breast cancer development in women.^19^ In addition, a systematic review on breast cancer risk factors in Iran revealed that high sugar consumption is a risk factor for breast cancer development, and daily exercise and vegetable consumption are protective factors against breast cancer development.^20^ Thus, the observed birth cohort effect may be explained in part by changing reproductive behaviors, as well as diet and lifestyle in Iranian women. Another explanation may be the fact that women in the recent cohorts have not yet reached their 40s and 50s when they become more likely to develop breast cancer.

 Studies on breast cancer have noted variable period and cohort effects across different geographic regions.^9^ For instance, in the US, age-standardized incidence rates of breast cancer declined from 1980 through to 2010,^21^ but increased in other countries during the same period.^22,23^ Most studies have shown that cohort effect is stronger than period effect, which may be attributed to reproductive risk factors,^9^ such as childbirth and breast feeding. Luo et al reported that in Guangzhou, China, in the period between 2004 and 2015, the incidence rate of female breast cancer increased in cohorts born before 1960 and then declined.^24^ Mubarik et al studied breast cancer incidence trends in four Asian countries (China, India, Pakistan, and Thailand) and found an overall increasing trend, with increased age and period effects, and declining birth cohort effect throughout the period 1990–2015. They also suggested that period effects influence breast cancer incidence rate through screening programs and disease management.^25^ It was suggested that the development of public health policies probably played an important role in the decreasing trend of breast cancer incidence in birth cohorts after the 1950s, although there are probably other factors not fully investigated yet.^25^ A study in Norway showed that between 1987 and 2008, the incidence rates of invasive breast cancer had steadily increased until 2002 with the highest rates among women aged 50–69, and then it started to decline from 2006.^26^ In a study in the Doubs region, France during the 1978–2003 period, a noticeable increase in invasive breast cancer incidence happened in women not undergoing routine mammography. Authors suggested that both cohort and period effects may have played an important role. The study showed a dramatically rising age effect until the age of 50 and a significant cohort effect with a peak for women born around 1940.^23^ In comparison with our results, we observed that the age effect peaked approximately a decade earlier at the age of 40 and the birth cohort effect occurred in the 1950s births.

 Several studies have suggested that the cohort effect is more obvious than the period effect.^23^ A study in Norway reported that 27% of cases of breast cancer in women aged 50-69 could be attributed to hormone treatment and approximately 23% of cases could be attributed to mammographic screening.^26^ The study also showed that both the initial and the subsequent screenings noticeably increased the incidence of detecting invasive breast cancer, while quitting the screening program resulted in decreased incidence rates.^26^ Several other studies have also suggested that screening programs and overdiagnosis substantially result in increased reporting of the incidence rates of breast cancer.^11,27^

 In conclusion, our findings indicated that the incidence of breast cancer peaks in the age of 45 in Iranian women, which is a decade earlier compared to the Western world. The period effect observed in this study can be explained by the fact that in 2014, the former Iranian pathology-based cancer registry was upgraded to a PBCR, which resulted in improved coverage of cancer patients. We also observed that the incidence rate of female breast cancer has declined in more recent birth cohorts, which might be because these women are younger and have not yet developed breast cancer.

 This study had several limitations. First, variation in the incidence rates of breast cancer may be attributed, to some extent, to the completeness of the INPCR over time in Iran population. The nationwide PBCR was developed in 2014. Before 2014, it was a pathology-based registry with noticeably limited coverage. Therefore, the incidence rates before 2014 may have been underestimated. In addition, the results of our study might have been affected by the rather short timeframe of the study. So further studies with longer duration and more longitudinal data are recommended to better estimate the effects of the APC. Despite these limitations, our study still can help explain the period and cohort effects on breast cancer incidence in Iran.
